# Identification of glycoproteins secreted by wild-type *Botrytis cinerea* and by protein *O*-mannosyltransferase mutants

**DOI:** 10.1186/s12866-014-0254-y

**Published:** 2014-10-12

**Authors:** Mario González, Nélida Brito, Celedonio González

**Affiliations:** U.D. Bioquímica y Biología Molecular, Universidad de La Laguna, 38206 La Laguna (Tenerife), Spain

**Keywords:** *Botyris cinerea*, Glyco-secretome, Mannosyltransferase

## Abstract

**Background:**

*Botrytis cinerea* secretes a high number of proteins that are predicted to have numerous *O*-glycosylation sites, frequently grouped in highly *O*-glycosylated regions, and analysis of mutants affected in *O*-glycosylation has shown, in *B. cinerea* and in other phytopathogenic fungi, that this process is important for fungal biology and virulence.

**Results:**

We report here the purification of glycoproteins from the culture medium, for a wild-type strain of *B. cinerea* and for three mutants affected in the first step of *O*-glycosylation, and the identification of components in the purified protein samples. Overall, 158 proteins were identified belonging to a wide diversity of protein families, which possess Ser/Thr-rich regions (presumably highly *O*-glycosylated) twice as frequently as the whole secretome. Surprisingly, proteins predicted to be highly *O*-glycosylated tend to be more abundant in the secretomes of the mutants affected in *O*-glycosylation than in the wild type, possibly because a correct glycosylation of these proteins helps keep them in the cell wall or extracellular matrix. Overexpression of three proteins predicted to be *O*-glycosylated in various degrees allowed to confirm the presence of mannose α1-2 and/or α1-3 bonds, but no mannose α1-6 bonds, and resulted in an enhanced activity of the culture medium to elicit plant defenses.

**Conclusions:**

Glycosylation of secretory proteins is very prevalent in *B. cinerea* and affects members of diverse protein families. *O*-glycosylated proteins play a role in the elicitation of plant defenses.

**Electronic supplementary material:**

The online version of this article (doi:10.1186/s12866-014-0254-y) contains supplementary material, which is available to authorized users.

## Background

*Botrytris cinerea* has been considered the second most important plant pathogenic fungus according to its economic and scientific importance [1], and is able to infect more than 200 plant species including many with high agronomic value [2]. The genome of two *B. cinerea* strains have been sequenced [3,4] and one of its key features is the prediction of a high number of secretory proteins, about 10% of the polypeptides coded by the genome. Proteomic studies have actually revealed an abundant and diverse set of proteins in the extracellular medium for *B. cinerea* cultures [5-11]. This set contains mainly proteins involved in the degradation of plant structures (such as cellulases, xylanases, proteases, etc.), but also proteins with other functions such as the induction of cell death in the host [12-14], as well as plenty of proteins with unknown function [7]. The role of some of these extracellular proteins has been studied by gene knock-out to identify secretome members contributing to virulence, i.e. virulence factors, but only a few have shown to contribute modestly to the infection process [14-19].

It is known that *O*-glycosylation is crucial in determining the structure and function of numerous secreted and membrane-bound proteins [20]. Carbohydrate chains have been proposed to enhance the stability and solubility of proteins, to confer protection against proteases, to act as a sorting determinant, and to be involved in various development and differentiation processes [20]. We have recently shown, by an *in silico* approach, that about half of predicted proteins in fungal secretomes display Ser/Thr-rich regions, i.e. regions with at least 40% Ser/Thr in a minimum of 20 contiguous residues [21], which are usually considered to display high-density of *O*-glycosylation [21-23]. Indeed, about one fourth of secretory proteins were predicted to display hyper-*O*-glycosylated regions, i.e. regions with at least 25% of residues predicted to be *O*-glycosylated [21]. In fungi, *O*-glycosylation begins with the addition of a mannose residue by protein *O*-mannosyltransferases (PMTs) in the lumen side of the endoplasmic reticulum (ER) membrane [24], a process which has recently been shown to occur, at least partially, concomitantly with the translocation of nascent polypeptides into the ER [25]. Three PMT families exist in fungi (PMT1, PMT2 and PMT4) [26,27], and filamentous fungi usually have only one *pmt* gene per family. The deletion of one or more of the *pmt* genes usually results in loss of viability or strong defects such as reduced conidia production, changes in fungal morphology, etc., emphasizing the importance of *O*-glycosylation for the fungal biology. The *B. cinerea* genome also contains three *pmt* genes [28], *bcpmt1, 2 and 4*, and the three of them, but especially *bcpmt2*, are critical for the stability of the cell wall, are necessary for sporulation, and are required for the generation of the extracellular matrix. Besides, BcPMTs are also required for full virulence in a variety of hosts, with a special role in the adhesion to, and penetration of, intact plant leaves [28].

Since PMTs have been found in both prokaryotes and eukaryotes [24], but not in plants, these proteins are promising targets in the design of novel control strategies against fungal phytopathogens. However, the ER-associated topology of these proteins [29] poses strong problems for the elucidation of their structure and the design of specific inhibitors. An indirect approach is the identification of specific PMT substrates that could also be important for fungal biology and virulence. Since we have previously observed significant changes in the patterns of protein secretion and glycosylation by the *bcpmt* mutants [28], we have addressed here the characterization of glyco-secretome differences between wild-type *B. cinerea* and *bcpmt* mutants.

## Results

### Many proteins in the *B. cinerea* secretome are glycosylated

**Figure 1:**
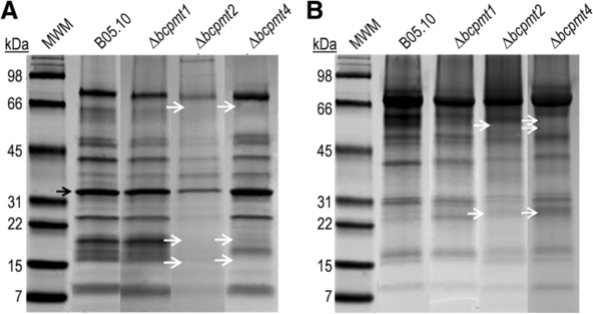
**Glyco-secretome purification.** Electrophoretic analysis of glycoproteins purified from the extracellular fraction in cultures of wild-type *B. cinerea* (B05.10) and the indicated Δ*bcpmt* mutants. **A**: SDS-PAGE showing all proteins precipitated from 150 μl of culture medium. **B**: SDS-PAGE of the purified glycoprotein samples (150 μl). Black arrow: BcAp8, a protein with no predicted glycosylation sites. White arrows: example bands appearing/disappearing in the mutant samples as compared with the corresponding wild-type sample.

We have previously identified more than one hundred proteins in the *B. cinerea* secretome [7], and we have also observed that the appearance of the secretome in 1D- and 2D-PAGE changes radically for mutants affected in PMTs, which catalyse the first step of *O*-glycosylation [28]. This prompted us to better characterize the set of glycoproteins secreted by wild-type *B. cinerea* strain B05.10, as well as by the mutants lacking each one of the three *B. cinerea* PMTs [28]. Preliminary observations allowed us to establish static liquid cultures in Petri dishes with YGG-low medium, inoculated with mycelial plugs and incubated for 4 days, as the optimum conditions to maximize isolation of secretory proteins in the case of Δ*bcpmt* mutants. Culture media obtained from these plates contained plenty of proteins (Figure 1A) and the band pattern in SDS-PAGE was different for the wild type and for the Δ*bcpmt* mutants. Purification of glycoproteins from the culture medium by affinity chromatography with Concanavalin-A resulted in quite different band patterns (Figure 1B), with some proteins clearly disappearing and others being enriched. Notably, the band corresponding to the most abundant protein in the secretome of *B. cinerea*, the 35-kDa aspartic protease BcAp8 [30] (black arrow in Figure 1A), is completely absent after purification of glycoproteins, in accordance with the fact that no *N*- or *O*-glycosylated sites are predicted *in silico* for this protein by the NetNGlyc 1.0 [31] and NetOGlyc 4.0 [32,33] servers. Moreover, an enrichment in proteins with high molecular weight (>50 kDa) was observed for all samples after glycoprotein purification, in agreement with the fact that lectin-blot experiments also show that glycosylation is more prominent for these proteins [28]. As expected, the SDS-PAGE band pattern of purified glycoprotein samples is different for the wild type and the three Δ*bcpmt* mutants, especially in the case of Δ*bcpmt2* and Δ*bcpmt4*.

**Figure 2:**
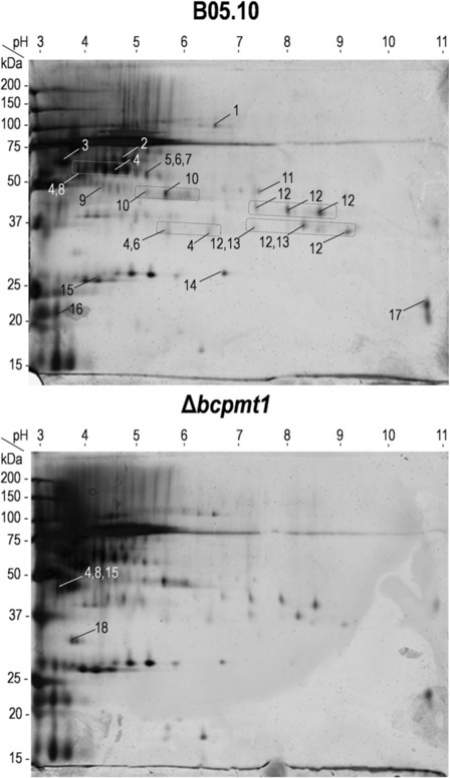
**2D Electrophoresis of the glyco-secretome.** 10 μg of purified protein sample were fractionated by 2D electrophoresis and stained with silver. Numbers indicate proteins identified in each spot by MALDI-TOF/TOF (Additional file 1). Boxes identify charge trains for which the same protein was identified in several spots.

2D electrophoresis of purified glycoproteins was carried out for B05.10 and the Δ*bcpmt1* mutant, and resulted in a relatively simple spot pattern (Figure 2), with not too many differences for the two samples. Some spots, however, showed clear differences in intensity, such as spot 18, which is over-expressed in the Δ*bcpmt1* sample. A total of 29 spots obtained from the wild-type or the Δ*bcpmt1* 2D gels were excised and analyzed by MALDI-TOF/TOF, resulting in the identification of 18 proteins (Additional file 1). In some cases (spots 4, 10, 12 and 13), the same protein was identified in several spots forming charge trains (different pIs), or even in spots (spots 4 and 12) with different mobility in the second dimension (different apparent molecular weight).

### Identification of glycoproteins in the secretome by LC-MS/MS

The purified glycoprotein samples obtained for the four strains were also analyzed by shotgun proteomics. The total number of proteins identified was 157 in the four samples (Additional file 1). Only one of the 18 proteins identified previously from the 2D electrophoresis gels (spot 13, Pectin methylesterase BcPME1) was not identified by LC-MS/MS, so the total number of glycoproteins identified by the two methods is 158. Most proteins identified (133 of 158) showed signal peptide according to SignalP 4.1 [34,35], and 22 others showed alternative secretion features according to SecretomeP 2.0 [36,37]. Forty-eight of the 158 proteins are described here for the first time as components of the *B. cinerea* secretome, while the rest have been reported previously [5-11].

Contrary to our initial expectations, the composition of the glyco-secretome was not radically different for the wild type and for the three Δ*bcpmt* mutants, so that proteins clearly absent in one or more of the mutants, and therefore putative substrates of the corresponding BcPMT, are scarce. Restricting the comparative analysis to highly expressed proteins (Table 1), so that the number of spectral counts accumulated for each protein makes the comparison more significant, it results that the 13 proteins considered are all expressed in the three Δ*bcpmt* mutants, as well as in the wild type. Only two proteins, pectin methylesterase BcPME2 and B0510_6786 (of unknown function), have an abundance of less than 25% that of the wild type in at least one of the mutants. Noticeably, some of these proteins are actually found at elevated levels in the mutants, in comparison with the wild type. Overall, these results confirm a prominent role for PMTs in protein glycosylation, but in a way that may be more complex than anticipated. It does not seem, for example, that individual proteins are substrates of only one BcPMTs.

**Table 1:** **Abundant proteins identified by LC-MS/MS in wild-type** ***B. cinerea*** **and the three Δ** ***bcpmt*** **mutants**

**Protein name** ^**1**^ **– Gene ID**	**Spectral counts** ^**2**^			
	**B05**	**Δ** ***bcpmt1***	**Δ** ***bcpmt2***	**Δ** ***bcpmt4***
1,3-beta-glucanosyltransferase Glycolipid anchored surface protein - B0510_3559	16	22	21	25
Alpha-amilase (GH13) - BC1G_02623.1	13	12	5	23
Beta-1,3-endoglucanase with a GPI anchor (GH17) - B0510_9551	5	15	6	17
Glucoamylase (CBM20, GH15) - B0510_2137	137	152	52	201
Glucoamylase (CBM20, GH15) - B0510_2884	30	39	15	34
Oxidorreductase - B0510_547	9	20	15	13
Pectin methylesterase BcPME2 (CE8) - B0510_344	31	37	9	4
Phospholipase C - B0510_184	11	17	14	15
Protease (Merops A1) - B0510_6952	7	18	8	12
Pro-Xaa carboxypeptidase (Merops S28) - BC1G_09564.1/BC1G_09565.1	16	23	7	15
Unknown, similar to phytase - B0510_5392	12	17	14	20
Unknown, similar to subtilase family protein - B0510_6786	21	22	4	14
Unknown, similar to yeast spore wall proteins - BC1G_10630.1	8	12	6	15

^**1**^Protein family and/or conserved domains found in sequences are included between brackets where possible: glycosyl hydrolase family (GH…), Merops family (Merops…), carbohydrate esterase family (CE…), or carbohydrate binding modules (CBM…).

^**2**^Only proteins fulfilling the following two conditions are displayed: they have at least 15 spectral counts in one of the four strains, and they account for more than 2% of all spectral counts in one of the strains.

**Figure 3:**
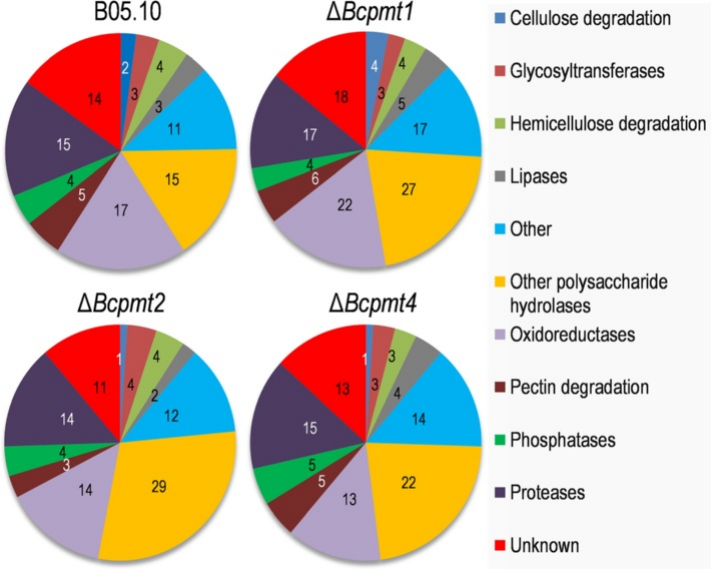
**Family distribution of the glycoproteins identified in the four** ***B. cinerea*** **strains.** Numbers indicate the number of proteins in each category.

The 158 proteins were classified into families previously defined [7] (Additional file 1). Family assignments were made using sequence similarities with proteins of known function detected by BLAST [38], and the presence of conserved domains according to Pfam [39,40]. Distribution of glycoproteins in families was similar for the wild type and the three mutants (Figure 3). The glyco-secretome is composed mainly of polysaccharide hydrolases (~36%), followed by proteases (~14%) and oxidoreductases (~13%). The rest of the families (~37%) are represented by a similar amount of proteins and contribute always less than 10% to the overall composition. As compared with the complete secretome [7], the glyco-secretome showed an increase in polysaccharide hydrolases and oxidoreductases, but a reduction in pectinases.

### Ser/Thr-rich and highly *O*-glycosylated regions in glycoproteins

**Figure 4:**
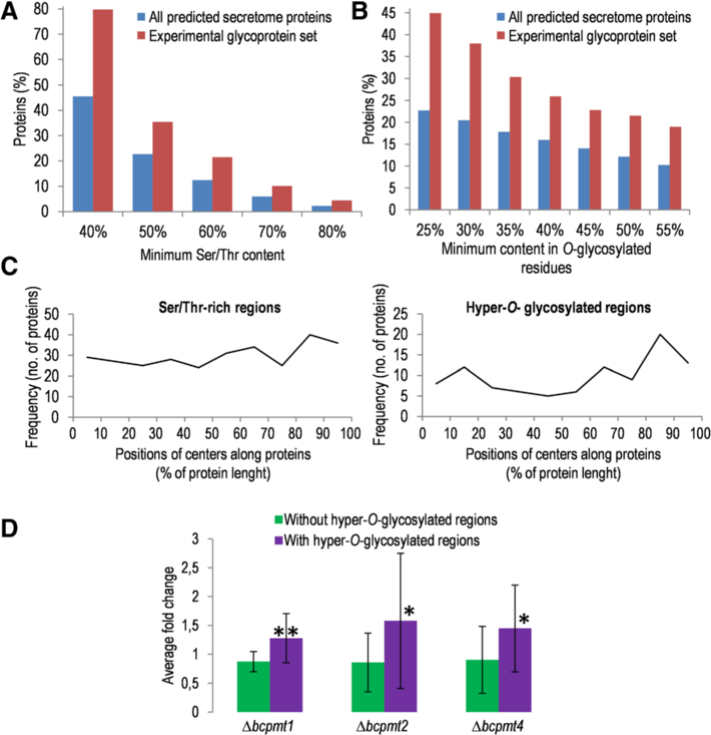
**Ser/Thr-rich regions and hyper-** ***O*** **-glycosylated regions among proteins in the glyco-secretome. A**: Percentages of proteins displaying regions with the indicated minimal Ser/Thr content (horizontal axis), both among the experimental glycoprotein set (red bars) and among all predicted secretome proteins (blue bars). **B**: Percentage of proteins with predicted hyper-*O*-glycosylated regions in the same two proteins sets, also analyzed for the indicated minimal abundances of predicted *O*-glycosylation sites (horizontal axis). **C**: Frequency distribution of Ser/Thr-rich and hyper-*O*-glycosylated regions along proteins for the experimental glycoprotein set. The total number of regions found in every possible 10% interval along protein length is represented. **D**: Relative abundance of proteins in the Δ*bcpmt* mutants, as compared with the wild-type strain B05.10, estimated from the spectral counts for two protein sets: those displaying hyper-*O*-glycosylated regions (purple bars) and those without them (green bars). Asterisks indicate a significant difference for the two sets (t-test) at either 0.95 (*) or 0.99 (**) confidence level.

We have recently analyzed *in sílico* the presence and distribution of Ser/Thr-rich regions, as well as hyper-*O*-glycosylated regions, in the predicted *B. cinerea* secretome [21]. We now have the opportunity to compare those results with a similar analysis for the experimental set of actually secreted and glycosylated proteins reported here (the 158 proteins displayed in Additional file 1). The results show, in first place, that Ser and Thr residues are extremely abundant among secreted glycoproteins. On average, 38% of residues in these proteins are either Ser or Thr in the experimental glycoprotein set, while this number is just 17% for the whole predicted secretome. When the MS Excel XRR macro described previously [21] was used to study if these residues are grouped forming Ser/Thr-rich regions (Figure 4A) a big number of these was found, even when a very high Ser/Thr content was necessary for a region to be considered as Ser/Thr-rich (up to 80%). The proportion of proteins displaying Ser/Thr-rich regions was considerably higher for the experimental set of glycoproteins than for the predicted whole secretome (Figure 4A). For example, 80% of glycoproteins displayed regions with at least 40% Ser/Thr, while only 46% of proteins in the whole predicted secretome exhibited them (Figure 4A). Moreover, the total number of Ser/Thr-rich regions was comparatively higher for the glycoprotein set: among the 158 glycoproteins 299 Ser/Thr-rich regions (with at least 40% Ser/Thr) were found, almost 2 per protein, while the whole predicted secretome (1910 proteins) contained only 1501 of these Ser/Thr-rich regions. Notably, seven proteins were found with regions of 20–32 residues in which at least 80% of them are Ser or Thr.

Similar results were obtained when glycosylation was predicted for the set of purified glycoproteins. Both *N*-glycosylation and *O*-glycosylation was predicted for most of them (91% and 87% of proteins, respectively), but the average number of predicted *O*-glycosylated residues in proteins was about three times higher than the number of *N*-glycosylated ones. The predicted *O*-glycosylation positions were frequently grouped in highly-*O*-glycosylated regions (Figure 4B), but not the *N*-glycosylation sites (not shown). Using the less stringent definition of highly-*O*-glycosylated regions (regions of at least 20 residues with 25% or more *O*-glycosylation sites, see ref. 21), it results that about half of proteins (45%) in the glyco-secretome display these kind of regions, about twice the number obtained for the whole predicted secretome (23%) [21].

We observed previously, for the whole predicted secretome, that Ser/Thr-rich and predicted highly *O*-glycosylated regions have a tendency to be located toward the two ends of polypeptide chains [21]. This tendency is clearer in the case of glycoproteins and especially evident for predicted highly *O*-glycosylated regions (Figure 4C), which are frequently found in the C-terminal region of proteins and are also common at the N-terminus right after the signal peptide.

Interestingly, we found a relationship between the presence of predicted hyper-*O*-glycosylated regions in proteins and their relative abundance in the secretomes of the Δ*bcpmt* mutants, estimated from the spectral counts. This was done by first choosing a set of abundant proteins, for which we could calculate more reliable fold changes from spectral counts, consisting of 29 proteins with at least 10 spectral counts in one of the four *Botrytis* strains and a relative abundance of at least 1% in one of the four strains. The fold change in the amount of protein in the Δ*bcpmt* mutants, relative to the wild type, was then estimated from the spectral counts for each individual protein. Next, the average fold change was calculated separately for those proteins with predicted hyper-*O*-glycosylated regions and for those without them, and are displayed in Figure 4D. Statistically significant differences were found for the two groups in the three mutants, so that the relative abundance of proteins predicted to have hyper-*O*-glycosylated regions is, on average, 1.43 times higher in the secretomes of the Δ*bcpmt* mutants than in the secretome of the wild type. On the contrary, no difference was observed for the proteins without predicted hyper-*O*-glycosylated regions.

**Figure 5:**
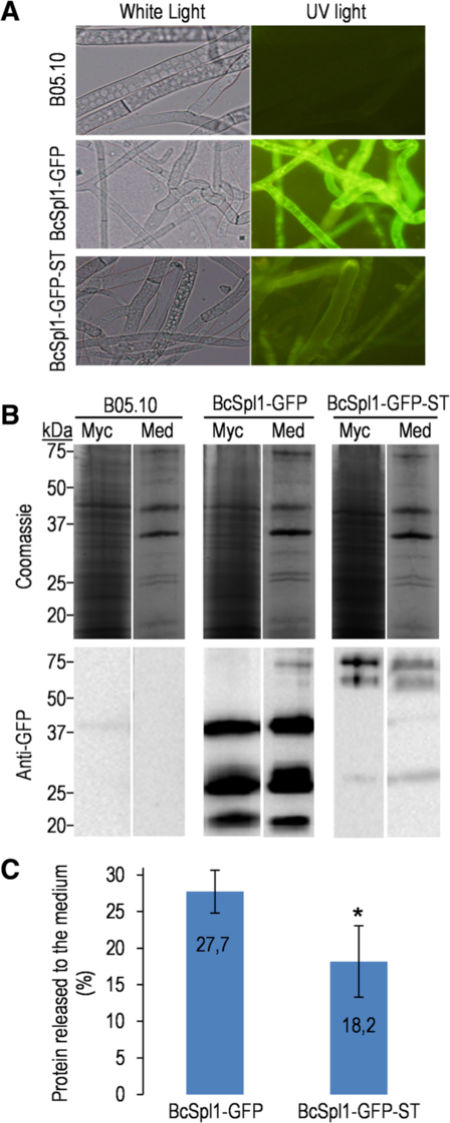
**Ser/Thr-rich regions partially retain proteins with the mycelium. A**: Fluorescence of transformants expressing a fusion proteins with BcSpl1, GFP, and with (BcSpl1-GFP-ST), or without (BcSpl1-GFP), a Ser/Thr-rich region from Cel5A. The wild-type strain B05.10 was used as control. **B**: SDS-PAGE and western blot (anti-GFP) comparing the amount of recombinant protein released to the culture medium (Med) and the amount associated with the mycelium (Myc) in cultures (16 hours in YGG-low medium) of the same strains as in **(A)**. Amounts of proteins loaded were those contained in 1.125 ml of medium (1/67 of total in culture) or associated with 7.5 mg of mycelium (1/122 of total in culture). **C**: Percentages of recombinant protein released to the medium as soluble protein for the two strains, averaged for three independent cultures. Asterisk indicates a statistically significant difference between the two strains with 0.95 confidence (Mann–Whitney test).

One possible explanation for the fact that proteins displaying hyper-*O*-glycosylated regions tend to be found at higher levels in the secretomes of the Δ*bcpmt* mutants is that *O*-glycosylation causes retention of the proteins in the cell wall or the extracellular matrix. These proteins, being glycosylated to a less extent in the Δ*bcpmt* mutants, would escape easier and accumulate to higher levels in the culture medium. To test this hypothesis, two fusion proteins were expressed in *B. cinerea* which both contained the sequence of BcSpl1 [14], an abundant component of the *B. cinerea* secretome partially retained in the cell wall [41], fused to GFP, but differed in the presence of a Ser/Thr-rich region in the C-terminal end originally coming from the endoglucanase Cel5A [42]. Unfortunately, the level of expression seemed quite different for the two fusion proteins, as judged from the green fluorescence in the mycelium (Figure 5A) and from the western-blots with monoclonal anti-GFP antibodies (Figure 5B), and both gave various bands in the western-blot. By considering all the bands obtained in the blots, we determined for liquid cultures of each of these two strains the ratio of protein secreted to the culture medium versus the amount associated with the mycelium (Figure 5C). Although the effect of the introduction of the Ser/Thr-rich tail in the protein was not spectacular, the fusion protein displaying it showed a higher tendency to be associated with the mycelium which was statistically significant in the three repetitions of the experiment shown in Figure 5C.

### Overexpression of *O*-glycosylated proteins in *B. cinerea*

Three of the proteins detected as components of the glyco-secretome were overexpressed in *B. cinerea* to further study them. These proteins were selected because they display Ser/Thr regions and are glycosylated in different degrees (Additional file 1). The three proteins were the endopolygalacturonase BcPG1, previously reported to be required for full virulence in *B. cinerea* [17] and to be perceived as a PAMP (Pathogen Associated Molecular Pattern) by *Arabidopsis* [43], and two proteins of unknown function that we have named BcIEB1 (B0510_2361, similar to IgE Binding proteins) and BcSUN1 (B0510_8844, similar to members of the yeast SUN family). These proteins differed in the number or length of the Ser/Thr-rich regions, with BcSUN1 being predicted to be highly glycosylated by NetOGlyc 4.0 and the other two poorly glycosylated (Additional file 1).

**Figure 6:**
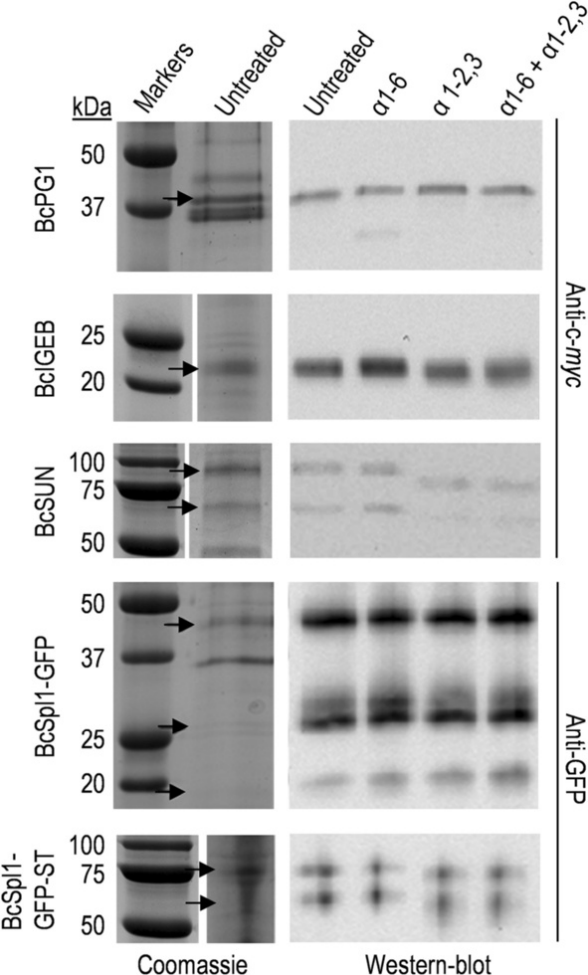
**Deglycosylation of** ***B. cinerea*** **proteins with exo α1-2,3 and exo α1-6 mannosidases.** Extracellular media from cultures of the strains overexpressing the indicated proteins were treated with the enzymes and analysed by western-blot with the indicated antibodies. SDS-PAGE of the untreated samples is also shown (Coomassie). The samples treated with the different enzymes, as well as the untreated controls, were identical and corresponded to 900 μl of culture medium. Arrows point to the positions in the Coomassie-stained gels that correspond to the bands observed in the western blots.

Expression of the three genes under the control of the strong promoter *oliC* resulted in the accumulation of decent amounts of proteins in the culture medium (Figure 6). In order to check if, and how, these proteins were glycosylated we treated them with two glycosyl hydrolases: exo α1-2,3 and exo α1-6 mannosidases. Since all attempts to purify them from the culture media were unsuccessful, the treatment was done with a whole secretome sample and analyzed by western-blot with anti-c-*myc* antibodies (Figure 6). Reduction of protein size, as a consequence of treatment, was considerable for BcSUN1 (estimated in 20–25 kDa), limited for BcIEB1 (estimated in 1–2 kDa), and not detectable for BcPG1, in good accordance with the number of glycosylation sites predicted for the three proteins: 74, 3 and 1 (Additional file 1). This reduction was seen only for the incubation with the exo α1-2,3 enzyme. Treatment with exo α1-6 mannosidase did not produced any effect by itself nor did it potentiate the effect of exo α1-2,3. These results indicate, therefore, that at least BcSUN1 and BcIEB1 are actually glycosylated, that the amount of sugars incorporated is in accordance with the predictions carried out by NetOGlyc, and that glycosidic chains contain primordially mannoses linked by α1-2 or α1-3 glycosidic bonds. Additionally, the same strategy was used to assess the incorporation of mannose residues to the BcSpl1-GFP-ST fusion protein described above (Figure 6), and also in this case treatment with exo α1-2,3 mannosidase (but not α1-6) produced a considerable reduction in size, but not in the case of the control fusion protein BcSpl1-GFP.

**Figure 7:**
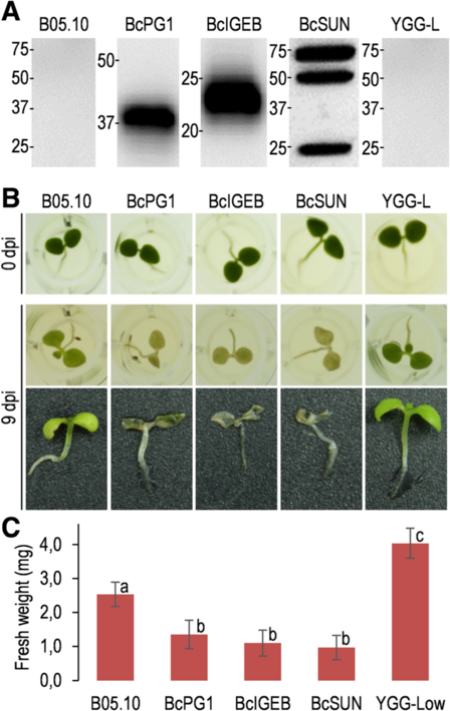
**Growth inhibition and necrosis of seedlings caused by** ***O*** **-glycosylated proteins.** Tobacco seedlings were treated for 9 days with culture media from the strains overexpressing the indicated *O*-glycosylated proteins and then assessed for necrosis and growth inhibition. **A**: Western-blot (anti-c-*myc*) showing the relative amounts of recombinant proteins in the culture media from the overexpressing strains. Medium from the wild-type strain (B05.10) and uninoculated medium (YGG-low) were used as controls. Each lane contained proteins precipitated from 1 ml of medium. Molecular weight makers are show to the left of each lane (kDa). **B**: Example seedlings treated with the culture media. **C**: Average weight (n=6) of treated seedling after the 9-day incubation. Different letters on bars indicate statistically significant differences with 0.99 confidence.

Culture media from the three strains overexpressing the *O*-glycosylated proteins were also tested for the ability to elicit defense responses in plants by seedling growth inhibition assay [44], a sensitive and quantitative test which correlates with typical plant defence responses such as callose deposition, production of reactive oxygen species, or pathogenesis-related gene expression [45,46]. In this assay, culture media from the three overexpressing strains revealed considerably more efficient in inhibiting growth of tobacco seedling, as compared with the media obtained with the wild-type strain (Figure 7A-C). Moreover, the seedlings treated with media from the overexpressing strains showed necrotic symptoms that were clearly more intense than with the wild-type strain.

## Discussion

*O*-glycosylated proteins are crucial components of fungal secretomes. Not only has it been predicted that more than half of secretome components display *O*-glycosylation sites [21], frequently in the form of highly-*O*-glycosylated regions, but it has also been shown that mutants affected in the first step of the *O*-glycosylation machinery display a vast arrays of defects including decreased virulence in the case of phytopathogens [28,47]. By purification and identification of glycoproteins, we have shown here that *B. cinerea* indeed secretes a substantial amount of these proteins. Altogether, 93 proteins were identified in the isolated glycoprotein sample for the wild-type strain B05.10, which include 18 proteins for which experimental evidence is provided here, for the first time, as components of the secretome. It is worth noting the absence, among these proteins, of the aspartic protease BcAP8, whose band is not detected in SDS-PAGE of the purified glycoprotein sample (Figure 1) nor does it appear among the 157 proteins identified by LC-MS/MS for the same sample. This protein serves as an internal negative control for the purification of glycoproteins, since it is the most abundant protein in the secretome [7] but it lacks predicted glycosylation sites. Its absence in the purified sample indicates that the purification is working properly. Family distribution of glycoproteins (Figure 3) is not very different from that obtained for the whole secretome sample [7], with a low number of proteins classes containing most of the proteins: mainly proteases, polysaccharide hydrolases, oxidoreductases, and plenty of proteins with unknown functions. It does not seem, therefore, that glycosylation acts preferentially on specific types of proteins.

Contrary to what we expected, the composition of the glyco-secretome was not very different for the wild type and the three Δ*bcpmt* mutants affected in *O*-glycosylation. We reasoned that lack of glycosylation of specific proteins in one or more of the Δ*bcpmt* mutants would prevent its retention by the lectin affinity column and so would result in its absence in the corresponding glycoprotein sample. Although there are plenty of proteins (62%) which are absent in one or more of the four strains, these correspond almost always to those with just one or a few spectral counts, so that their absence/presence may be the result of mere chance. The comparison of spectral counts for those proteins with the highest expression (Table 1), however, does not show any protein which is absent in the mutant samples. This may indicate, at least for these proteins, that they are not substrates of single PMTs (or PMT dimers) but are glycosylated by the combined action of several of them. PMTs have been shown to act as specific dimers in *Saccharomyces cerevisiae*, Pmt1/Pmt2 heterodimer and Pmt4/Pmt4 homodimer [48], and as almost all possible hetero- and homo-dimeric combinations in *Aspergillus nidulans* [49]. Our results agree better with the situation in *A. nidulans*, since a more promiscuous association of PMT monomers, to form functional dimers, would inherently imply an easier substitution of one of the PMTs by the other isoforms.

Surprisingly, more often than not, spectral counts for the most abundant proteins are higher in the Δ*bcpmt* mutants than in the wild-type strain B05.10 (Table 1), possibly because proteins correctly glycosylated are retained easier in the cell surroundings (cell wall and/or extracellular matrix) while those with an incomplete glycan structure escape easier. It was actually shown (Figure 4D) that proteins predicted to have hyper-*O*-glycosylated regions are, on average, more abundant in the glyco-secretomes of the Δ*bcpmt* mutants, as compared with the wild type, while the same is not true for the proteins not predicted to have hyper-*O*-glycosylated regions. Moreover, addition of a Ser/Thr rich region to a fusion protein expressed in *B. cinerea* increases the amount of it that remains associated with the mycelium (Figure 5), as compared with the amount found in the extracellular medium, although these results need to be taken with caution because of the different levels of GFP expression in the two strains. If confirmed, this effect of glycosylation maybe physiologically important for *B. cinerea*, as enzymes acting on soluble substrates and producing assimilable nutrients for the fungal cells may be more efficient if retained closer to the cells.

The overexpression of three of the proteins predicted to be *O*-glycosylated allowed experimental confirmation of the post-translation modification for two of them, as a decrease in the apparent molecular mass observed in SDS-PAGE was caused by enzymatic deglycosylation (Figure 6). Although prediction of *O*-glycosylation carried out by NetOGlyc is only approximate for fungal proteins [21], the changes in the apparent molecular mass obtained for the three proteins are in accordance with the number of *O*-glycosylation sites predicted (74, 3, and 1 for BcSUN1, BcIEB1, BcPG1, respectively). Incubation with two different deglycosylation enzymes showed the presence of mannose α1-2 and/or α1-3 bonds (Figure 6), but no mannose α1-6 bonds were detected. These results are in agreement with the glycosyl linkages observed for other fungal and yeast proteins, usually displaying mannose α1-2 and α1-3 linkages [20]. Although mannose α1-6 bonds have been observed in other fungi such as *Aspergillus* [20], they either do not exist in *B. cinerea*, at least in the proteins analyzed, or their contribution to the proteins molecular weight is not significant.

The extracellular media obtained after growing the strains overexpressing the three glycoproteins showed an enhanced ability to elicit plant defenses, as detected in seedling growth inhibition assays (Figure 7). This was not surprising in the case of BcPG1, since this proteins has recently been shown to be recognized as a PAMP by the *Arabidopsis* pattern recognition receptor RBPG1 [43], but was completely unexpected for the other two. It is tempting to speculate that it is the sugar fraction on these proteins the part responsible for the elicitation of the plant defenses, since this feature is shared by the three proteins, but clearly the alternative explanation, i.e. different amino acid sequences in the three proteins act as elicitors, cannot be ruled out at this point.

## Conclusions

*B. cinerea* secretes plenty of glycosylated proteins belonging to a diverse set of families, with polysaccharide hydrolases, proteases, and oxidoreductases being the most abundant groups. As expected, Ser/Thr-rich regions, considered to be substrates of the *O*-glycosylation machinery, are twice more abundant in the glyco-secretome than in the whole secretome. Surprisingly, proteins predicted to be hyper-*O*-glycosilated are more abundant in the glyco-secretomes of *O*-glycosylation deficient mutants, possibly because *O*-glycosylation causes retention in the cell wall or extracellular matrix. *O*-glycosylated proteins seem to have a prominent role in plant-pathogen interaction, since the independent overexpression of three of them in *B. cinerea* increases elicitation of plant defenses by the fungus.

## Methods

### Strains and growth conditions

*B. cinerea* strains B05.10 [50] and the three *bcpmt* knock-out mutants (Δ*bcpmt1*, Δ*bcpmt2*, and Δ*bcpmt4*) [28] were maintained as conidial suspensions, or as mycelium in agar plugs for strain Δ*bcpmt2* (it does not produce conidia), in 15% glycerol at −80°C for long storage. For routine use, fungal strains were maintained at 4°C in silica gel [51]. Unless otherwise indicated, *B. cinerea* was grown in YGG-low medium (0.5% Yeast Extract, 10 mM glucose, and 0.3% Gamborg’s B5), inoculated with 10^6^ conidia per ml, at 20°C with shaking at 150 rpm. A description of all *B. cinerea* strains used in this work can be found in Table 2.

**Table 2:** ***B. cinerea*** **strains used in this work**

***Botrytis cinerea*** **strain**	**Description**	**Antibiotic resistance**
B05.10	Wild-type strain of *B. cinerea* [50]	None
Δ*Bcpmt1*	*bcpmt1* gene knock-out mutant	Hygromycin-B resistance [28]
Δ*Bcpmt2*	*bcpmt2* gene knock-out mutant	
Δ*Bcpmt4*	*bcpmt4* gene knock-out mutant	
B05.10 (BcSpl1-GFP)	Transformant of B05.10, expressing a fusion protein with BcSpl1 [14], GFP [58], and tags (6xHis; c-*myc*)	
B05.10 (BcSpl1-GFP-ST)	Transformant of B05.10, expressing a fusion protein with BcSpl1 [14], GFP [58], a Ser/Thr-rich region from Cel5A [42] and tags (6xHis; c-*myc*)	
B05.10 (BcPG1)	Transformant of B05.10, over-expressing the *B. cinerea* protein BcPG1, with tags (6xHis; c-*myc*)	Nourseothricin resistance. The expression construction was integrated at the *niaD* (nitrate reductase) locus [57].
B05.10 (BcIEB1)	Transformant of B05.10, over-expressing the *B. cinerea* protein BcIEB1, with tags (6xHis; c-*myc*)	
B05.10 (BcSUN1)	Transformant of B05.10, over-expressing the *B. cinerea* protein BcSUN1, with tags (6xHis; c-*myc*)	

### Isolation and quantification of extracellular glycoproteins

Secretome samples were obtained from fungal cultures in Petri dishes. Usually, ten plates with 20 ml of YGG-low medium were inoculated with mycelial plugs of the indicated strains and incubated 4 days at 20°C in the dark, without shaking. The culture medium was then harvested by filtration through 4 layers of filter paper and frozen at −20°C until use. After thawing, glycoproteins were purified by affinity chromatography on Concanavalin-A Sepharose. The culture medium (approx. 100 ml) was first equilibrated with 1/3 volume of 4X binding buffer (80 mM Tris–HCl pH 7.4, 2 M NaCl, 4 mM MnCl_2_, 4 mM CaCl_2_), centrifuged (1 000×g, 10 min) to remove insoluble material, and run through a Hi-Trap Con-A 4B prepacked column (GE Healthcare 28-9520-85) at a flow rate of 1 ml/min. Proteins binding non-specifically to the column were removed by washing with 15 ml of 1X binding buffer at the same flow rate. Glycoproteins were finally eluted with elution buffer (1X binding buffer supplemented with 300 mM methyl-α-D-glucopyranoside), at a flow rate of 0.5 ml/min, and 1-ml fractions were collected. Protein concentration was determined by the method of Bradford [52], and usually fractions 2–4 contained appreciable protein concentration and were pooled. Typically, about 25–110 μg of glycoproteins were obtained, with the higher quantity corresponding to the wild-type strain B05.10.

### Protein electrophoresis and identification

Unless otherwise indicated, protein samples were concentrated for electrophoresis by precipitation with methanol-chloroform [53] followed by resuspension in SDS-PAGE sample buffer. SDS-PAGEs were carried out on Any-KD Mini-PROTEAN TGX precast gels (Bio-Rad), and the gels were stained with colloidal Coomassie brilliant blue [54]. 2D electrophoresis were carried at the CNB Proteomics Facility (Centro Nacional de Biotecnología, Madrid, Spain), stained with silver, and the proteins in the indicated spots were then identified as explained elsewhere [28].

Protein identification by LC-MS/MS was carried out at the proteomics facility of the Centro de Biología Molecular Severo Ochoa (Madrid, Spain). Samples of glycoproteins were precipitated with methanol-chloroform, dried, and the pellet sent to the facility to be redissolved in SDS-PAGE sample buffer and analyzed by LC-MS/MS, basically as reported by Clemente *et al.* [55], but using a different database, uniprot-fungi.fasta, for peptide identification. False discovery rate was lower than 0.01.

### Bioinformatics

Signal peptide detection was performed using the SignalP 4.1 server [34,35]. The prediction of non-clasical protein secretion was done with the SecretomeP 2.0 server [36,37], using default parameters. Family assignment was done using sequence similarities to proteins of known function detected by BLAST [38,56], and the presence of conserved domains reported by Pfam [39,40]. Those proteins with no high similarity to proteins with known function were labelled as dubious, but the BLAST best hit, or the Pfam family and/or conserved domains, are also reported. In these cases, the proteins are included in the corresponding family, as defined by BLAST or Pfam. Only the proteins without any clear BLAST similarity or conserved domains are included in the “Unknown” family.

In order to search for Ser/Thr-rich regions in proteins, as well as regions predicted to be hyper-*O*-glycosylated, we used the Microsoft Excel macro XRR previously described [21]. Positions of Ser/Thr residues along every protein, and/or positions of predicted *O*-glycosylation sites identified by NetOGlyc 4.0 [32], were transferred to an Excel spreadsheet and loaded into the XRR macro. The XRR macro basically reports a list of regions with a length of at least of 20 amino acids displaying the indicated minimum content of Ser + Thr (or predicted *O*-glycosylation sites). In order to plot the distribution of the Ser/Thr-rich regions along proteins, the central positions of all Ser/Thr-rich regions was calculated as percentage distance from the N-terminus and grouped in ten categories (0-10%, 10-20%, …), as described before [21].

### Expression of recombinant proteins in *B. cinerea*

PCR amplifications were made with Phusion High-Fidelity DNA Polymerase (New England Biolabs) when the DNA product was to be used in cloning experiments, and *Taq* polymerase (GenScript) was used in any other case. All oligonucleotides used (Additional file 2) were from Life Technologies. Yeast recombinational cloning (YRC) was carried out as described by Schumacher [57], and plasmid extraction from *Saccharomyces cerevisiae* was made with the E.Z.N.A. Yeast Plasmid Kit (OMEGA bio-tek).

A fusion protein composed of GFP, the *B. cinerea* cerato-platanin BcSpl1, and a Ser/Thr-rich region from the *B. cinerea* protein Cel5A (a putative substrate for *O*-glycosylation) [42], was expressed in *B. cinerea* by transformation with plasmid pNDN-GFP-ST (see below). A control protein lacking the Cel5A Ser/Thr-rich region was expressed with plasmid pNDN-GFP. Plasmid pNDN-GFP was constructed by YRC introducing in plasmid pNDN-OGG [57], linearized with *Nco*I, a DNA fragment amplified from plasmid pCRP-GFP [41] with primer pair pCRPOGG-FW/pCRPOGG-RV and displaying i) the complete ORF of the *bcspl*1 gene [14], ii) the c-*myc* and 6xHis epitopes (originally from pPICZαA; Invitrogen, Carlsbad, CA, USA), and iii) a codon-optimized GFP gene, adapted for *B. cinerea* and originally coming from plasmid pOptGFP [58]. To obtain the pNDN-GFP-ST plasmid, a 299-bp DNA fragment containing the Ser/Thr-rich region from the endo-ß-1,4-glucanase Cel5A [42] was amplified with the primer pair pCRPOGGST-FW/pCRPOGGST-RV, using *B. cinerea* B05.10 genomic DNA as template, and cloned into *Not*I-linearized pNDN-GFP by YRC.

Three *B. cinerea* proteins, BcPG1 (acc. no. B0510_5388) [17], BcIEB1 (acc. no. B0510_2361) and BcSUN1 (acc. no. B0510_8844) were overexpressed in *B. cinerea* B05.10, fused to various epitopes, by transformation with plasmids pCBN-EPG, pCBN-IGE and pCBN-SUN, respectively, which were constructed as follows. An intermediate plasmid, pCBN, was first assembled by fusing with YRC the following three fragments: i) a 6583-bp fragment amplified from plasmid pBHT2 [59] with the primer pair CAMBIA-FW/CAMBIA-RV, ii) a 3452-bp fragment amplified from plasmid pNDN-OGG [57] with the primer pair NDN-CAMBIA-FW/NDN-CAMBIA-RV and digested with *Eco*RI and *Hind*III, and iii) a 4175-bp fragment also amplified from pNDN-OGG but with primer pair NDN-FW/NDN-RV. In a second step, two inserts were cloned simultaneously by YCR in pCBN previously digested with *Nco*I plus *Hind*III: i) a 121-bp fragment coding for the 6xHis and c-*myc* eptitopes amplified from pPICZαA with primer pair CMYC-FW/CMYC-RV, and ii) one of the three genes coding *B. cinerea* proteins. The latter were obtained by PCR, using *B. cinerea* B05.10 genomic DNA as template and the following primer pairs: EPG1-FW/EPG1-CMIC-RV for pCBN-EPG (expression of BcPG1), IGE-FW/IGE-CMIC-RV for pCBN-IGE (expression of BcIEB1), and SUN-FW/SUN-CMIC-RV for pCBN-SUN (expression of BcSUN1).

*B. cinerea* transformations were carried out as described by Hamada et al. [60], with the modifications introduced by van Kan et al. [61]. The five plasmids (pNDN-GFP, pNDN-GFP-ST, pCBN-EPG, pCBN-IGE and pCBN-SUN) were transformed into *B. cinerea* by integration in the *niaD* locus [57]. The following fragments from the plasmids were used in the transformations: *Sac*I-*Apa*I fragment from pNDN-GFP (5605 bp) or pNDN-GFP-ST (5842 bp), *Not*I-*Pst*I fragment from pCBN-EPG (7398 bp) or pCBN-IGE (6868 bp), and *Not*I-*Xba*I fragment from pCBN-SUN (6898 bp). These DNA fragments were all purified from agarose gels after digestion.

Transformants were analyzed by PCRs to check for the right integration events at the *niaD* locus, with primer pairs binding to the transforming DNA, on one side, and to a *niaD* region not included in the transforming DNA, on the other (see Additional file 2 for details). Homokaryosis was confirmed in every transformant by ensuring the absence of the wild-type *niaD* gene with PCR (see Additional file 2). All transformants used in the rest of the work gave the expected results in these PCRs (not shown).

### Detection and quantification of recombinant proteins

The secretomes of the *Botrytis* transformants were analyzed by SDS-PAGE and western-blots using nitrocellulose membranes (Whatman Protran BA 85) and monoclonal anti-GFP or anti-c-*myc* primary antibodies (Roche) diluted 1:1000. Secondary antibodies consisted of goat anti-mouse IgG conjugated to Horseradish peroxidase (Sigma-Aldrich) and were used at a 1:3000 dilution. The peroxidase was detected with Immobilon Western Chemiluminescent HRP Substrate (Millipore). Quantification of western-blot bands was done with the software Quantity One (Bio-Rad) on the chemiluminescence signal recorded with a Gel Doc XR + system (Bio-Rad).

### Seedling growth inhibition experiments

Samples to be tested in these assays consisted in culture media obtained after growing *B. cinerea* for 16 hours in YGG-low medium, inoculated with 3 · 10^6^ conidia/ml. These media were filtered with 4 layers of filter paper and frozen until use. Thawed culture media were first centrifuged (20 min, 16 000xg) and 200 μl were then added into 96-well microtiter plates and incubated with *Nicotinana tabacum* cv. Havana seedlings. After 6–8 days in a phytotron, plants were photographed and weighted. To obtain the plants, seeds were sterilized with chlorine gas [62] and germinated in Petri dishes with 20 ml of half-strength Murashige-Skoog medium (Pronadisa, Spain) for 20–30 days.

### Exo-mannosidase treatment

Recombinant exo-mannosidases, α1-6 mannosidase (P0727S) and α1-2,3 mannosidase (P0729S), were from New England Biolabs (Ipswich, Massachusetts). Proteins in 1 ml of culture medium were precipitated with methanol-chloroform [53], resuspended in water, and incubated with 40 units of α1-2,3 and/or 32 units of α1-6 mannosidases under standard conditions recommended by the supplier. Reactions were first incubated for 18 hours at 37°C, then the same amounts of enzyme/s were added and incubation was prolonged for 2 more hours. Reactions were stopped by the addition of SDS-PAGE sample buffer.

## Additional files

Additional file 1:
**Protein content of the** ***Botrytis cinerea*** **glyco-secretome.**
Additional file 2:
**Oligonucleotides used in this study.**
