# Supplementary material for: Identification of glycoproteins secreted by wild-type Botrytis cinerea and by protein O-mannosyltransferase mutants
Source: BMC Microbiol. 2014 Oct 12;14:254. doi: 10.1186/s12866-014-0254-y (PMC4197228; doi:10.1186/s12866-014-0254-y)
Supplement: Additional file 2: — Oligonucleotides used in this study. [file 12866_2014_254_MOESM2_ESM.pdf]

## **Additional file 2**. Oligonucleotides used in this study

| Primer ID. | Seq (5' → 3') | Comments |
|---|---|---|
| pCRPOGG-FW | TCCATCACATCACAATCGATCCAACCATGCAATTCCCAACTCTCG | Used to amplify a 542-bp DNA fragment from plasmid pCRP-GFP (see text). pCRPOGG-FW (in combination with NR-RV-check) was also used to check for the right integration event of plasmids pNDN-GFP and pNDN-GFP-ST at the *NiaD* locus. |
| pCRPOGG-RV | TACTTACCTCACCCTTGGAAACCATGGTGAACCCGGGATGATG | |
| pCRPOGGST-FW | TGGGAATGGATGAACTTTACAAAGCGACCACTTCCGCTGCCGTTG | Used to amplify a 293-bp DNA fragment, which codes for the Ser/Thr-rich region of the Cel5A, from *B. cinerea* B05.10 genomic DNA (see text). |
| pCRPOGGST-RV | TCATACATCTTATCTACATACGCTATCCGGCAGCTGTGCTGCC | |
| CAMBIA-FW | TGGCAGGATATATTGTGGTGTAAACAGAATTCGAGCTCGGTACCCG | Used to amplify a 6583-bp DNA fragment from plasmid bpHT2, used to generate the vector pCBN by YRC (see text). |
| CAMBIA-RV | CCAGCCAGCCAACAGCTC | |
| NDN-CAMBIA-FW | TGCCGGTCGGGGAGCTGTTGGCTGGCTGGTGACTGGGAAAACCCTGGC | Used to amplify a 3452-bp DNA fragment from plasmid pNDN-OGG, used to generate the vector pCBN by YRC (see text). |
| NDN-CAMBIA-RV | TGTTTACACCACAATATATCCTGCCACAAATAGGGGTTCCGCGC | |
| NDN-FW | TTTGTGGCAGGATATATTGTGGTGTAAACAGCCGATTCATTAATGCAGC | Used to amplify a 4175-bp DNA fragment from plasmid pNDN-OGG, used to generate the vector pCBN by YRC (see text). |
| NDN-RV | CTAATAAACGCTCTTTTCTCTTAGGTTTACTTCACGACAATAGCACGGAC | |
| CMYC-FW | TCACATCACAATCGATCCAACCATGGAACAAAAACTCATCTCAG | Used to amplify a 121-bp DNA fragment, which codes for c-*myc* and 6xHis epitopes, from plasmid pPICZαA (see text). |
| CMYC-RV | TAATCATACATCTTATCTACATACGAAGCTTTCAATGATGATGATGATGATGG | |
| EPG1-FW | TCCATCACATCACAATCGATCCAACCATGGTTCAACTTCTCTCAATGGC | Used to amplify a 1196-bp DNA fragment, which contains the complete ORF of the *bcpg1* gene, from *B. cinerea* B05.10 genomic DNA. EPG1-FW (in combination with NR-RV-check) was also used to check for the right integration event of plasmid pCBN-EPG at the *NiaD* locus. |
| EPG1-CMIC-RV | CCTCTTCTGAGATGAGTTTTTGTTCACACTTGACACCAGATGGGAG | |
| IGE-FW | TCCATCACATCACAATCGATCCAACCATGTTCTCCAAGACCTTCATCG | Used to amplify a 666-bp DNA fragment, which contains the complete ORF of *bcieb1* gene, from *B. cinerea* B05.10 genomic DNA. IGE-FW (in combination with NR-RV-check) was also used to check for the right integration event of plasmid pCBN-IGE at the *NiaD* locus. |
| IGE-CMIC-RV | CCTCTTCTGAGATGAGTTTTTGTTCAGCGTACTCCCAAGCGGAAGGGC | |
| SUN-FW | TCCATCACATCACAATCGATCCAACCATGAAGTTCACCCCAGTTTC | Used to amplify a 1519-bp DNA fragment, which contains the complete ORF of *bcsun1* gene, from *B. cinerea* B05.10 genomic DNA. SUN-FW (in combination with NR-RV-check) was also used to check for the right integration event of plasmid pCBN-SUN at the *NiaD* locus. |
| SUN-CMIC-RV | CCTCTTCTGAGATGAGTTTTTGTTCTGATGAGAAGACGTATGTAGCG | |
| NOUR-RV-check | CAGGCGCTCTACATGAGC | Used to check for the right integration event of plasmids pNDN-GFP, pNDN-GFPST, pCBN-EPG, pCBN-IGE and pCBN-SUN at the *NiaD* locus. NOUR-RV-check binds inside of the transforming DNA region, at the *Nat1* gene not present in the recipient strain. NR-FW-check binds to a *NiaD* promoter region not present in any of the plasmids. |
| NR-FW-check | AGGATGGTTTGGTTTCGG | |
| NR-RV-check | GTCTGAGATAGCCCTACGG | Used to check for the right integration event of plasmids pNDN-GFP, pNDN-GFP-ST, pCBN-EPG, pCBN-IGE and pCBN-SUN at the *NiaD* locus, in combination with the specific primers pCRPOGG-FW, EPG1-FW, IGE-FW and SUN-FW. NR-RV-check binds to a *Nia*D terminator region not present in any of the plasmids. |
| NR-MBD-SMA | TCCCCCGGGTTTACCATGATCGGGTCGA | Used to check the absence of the *NiaD* gene in transformants obtained by integration at the *niaD* locus, in order to ensure homokaryosis. |
| NR-MBD-BAM | CGGGATCCACCTCCTTTAAGCGCTTT | |
